# Prevalence and characteristics of positional obstructive sleep apnea in a Saudi population-based cohort

**DOI:** 10.1097/MD.0000000000040455

**Published:** 2024-11-15

**Authors:** Siraj Wali, Omar Kanbr, Faris Alhejaili, Ranya Alshumrani, Md Dilshad Manzar, Haneen Mansour

**Affiliations:** a Sleep Medicine Research Group, Sleep Medicine and Research Center, King Abdulaziz University Hospital, Jeddah, Saudi Arabia; b Respiratory Section, Department of Medicine, Faculty of Medicine, King Abdulaziz University, Jeddah, Saudi Arabia; c Faculty of Medicine, Elrazi University, Khartoum, Sudan; d Respiratory Division, Pediatric Department, Faculty of Medicine, King Abdulaziz University, Jeddah, Saudi Arabia; e Department of Nursing, College of Applied Medical Sciences, Majmaah University, Al Majmaah, Saudi Arabia.

**Keywords:** exclusive positional obstructive sleep apnea, positional obstructive sleep apnea, prevalence, Saudi Arabia, sleep-disordered breathing

## Abstract

Obstructive sleep apnea (OSA), significantly impacts public health, with varying prevalence rates across populations. Positional obstructive sleep apnea (POSA) is a subtype in which OSA predominantly occurs in the supine position. This study investigated the prevalence and characteristics of exclusive positional obstructive sleep apnea (e-POSA) in a representative Saudi population. Data from a previous cohort study on the prevalence of OSA in Saudi Arabia were utilized. A 2-phase approach was used: the first phase involved a screening questionnaire, and the second phase involved confirmatory polysomnography (PSG). E-POSA was defined as an apnea-hypopnea index (AHI) in the supine position at least twice as high as that in the lateral position, with the lateral AHI not exceeding 5. Then, an exploration method was used to estimate the prevalence of e-POSA. There were 235 OSA patients included in this study. The prevalence of e-POSA among the OSA patients was 21.28%, with a higher prevalence in females (26.76%) than in males (18.90%). However, the estimated prevalence of e-POSA in the Saudi population was 1.86%. Patients with e-POSA tended to be younger and to have lower AHI, Epworth Sleepiness Scale (ESS) and arousal index scores (*P* < .001). Multivariate analysis revealed that the rapid eye movement (REM) related AHI and nonsupine AHI were predictors of e-POSA (*P* < .01). E-POSA is common in patients with OSA. However, the estimated prevalence of e-POSA across the general population was 1.86%. Patients with e-POSA have milder disease, and the AHI-REM and AHI-nonsupine were identified as predictors.

## 1. Introduction

Sleep-disordered breathing (SDB) encompasses a spectrum of sleep disorders characterized by abnormal breathing during sleep. Obstructive sleep apnea (OSA) is the most common type of sleep-disordered breathing worldwide and is characterized by repeated episodes of a complete (apnea) or partial collapse (hypopnea) of the upper airway with an associated decrease in oxygen saturation and arousal from sleep.^[1]^ This condition is usually accompanied by disruptive snoring and excessive daytime sleepiness (EDS). A high prevalence of OSA was recently reported, with OSA affecting 23% of middle-aged women and up to 49% of middle-aged men.^[2]^ However, in Saudi Arabia, 12.4% of men and 4.8% of women are estimated to have clinically significant OSA.^[3]^

Failure to address OSA can have severe repercussions. OSA can begin with EDS, potentially resulting in critical road traffic accidents.^[4]^ Furthermore, if left untreated, OSA can escalate to more severe health issues, including cardiovascular conditions. Severe and untreated OSA may be associated with high morbidity and mortality.^[5]^ Importantly, in the majority of cases, OSA can be easily treated. Seeking timely medical attention and adhering to prescribed treatments can significantly mitigate these risks.

Different subtypes of OSA, including positional obstructive sleep apnea (POSA), which refers to OSA that occurs mainly in the supine position, can be identified via polysomnography (PSG). Although there are several definitions of POSA in the literature,^[6]^ the strictest definition is an apnea-hypopnea index (AHI) score in the supine position at least twice as high as that in the lateral position,^[7]^ with exclusive positional obstructive sleep apnea (e-POSA) defined as an AHI score in the lateral position not exceeding 5.^[8]^

Although the prevalence of POSA reported in the literature varies widely based on the POSA definition used, a recent study reported a high prevalence rate of POSA (75%) in OSA patients in comparison to that of e-POSA, which reached up to 36%.^[2]^ Furthermore, the prevalence of POSA in a sample of the Saudi population was reported to be 54% among all OSA patients, but that of e-POSA was 38.6%.^[6]^ Nevertheless, only 1 study in the literature has examined the prevalence of POSA in the general population. Heinzer et al^[9]^ reported that POSA occurs in 53% of the general population and that e-POSA occurs in only 26% of the general population.

Other epidemiological studies, including local studies, have relied on samples of individuals who underwent PSG within their specific centers and primarily for diagnostic indications. Although valuable, this study method may not be ideal for accurately representing the entire population. Hence, in this study, we employed a rigorous approach that is better suited for providing more comprehensive insight into the prevalence of these conditions and representing our population.

Distinguishing e-POSA from other types of OSA is crucial not only for applying targeted therapies but also for improving patient outcomes and quality of life. Positional therapy, which involves strategies to prevent patients from sleeping on their back, has been shown to be an effective treatment option for a significant proportion of patients with e-POSA. Studies have indicated that patients with mild to moderate e-POSA can effectively manage their condition with positional therapy alone,^[10]^ highlighting the potential for less invasive, cost effective and high-compliance treatment options compared to conventional methods such as continuous positive airway pressure (CPAP) therapy or surgical interventions. Subsequent studies have reinforced these findings, showing that positional therapy can lead to significant improvements in AHI scores, oxygen saturation levels, and overall sleep quality for individuals with e-POSA.^[7]^ This approach not only provides an alternative therapy for patients who are intolerant to CPAP therapy but also emphasizes the importance of comprehensive sleep assessments that include positional analysis to tailor the most effective and least invasive treatment plans.^[9]^

This study is the first to determine the estimated prevalence of POSA using data from a representative sample of the Saudi population, which was previously part of a cohort study used to determine the prevalence of OSA in Saudi Arabia.

## 2. Methodology

### 2.1. Participants

All participants from a Saudi population study, as previously described, were included in this analysis to determine the prevalence of OSA.^[3]^ Briefly, this epidemiological study was a population-based study, and the target population comprised Saudi school employees aged 30 to 60 years, including porters, drivers, teachers and administrators from randomly chosen schools.

The study was performed in 2 stages. The first stage was a screening stage: a random sample of Saudi employees (n = 2682) aged 30 to 60 years completed a survey that included the Wisconsin questionnaire. EDS was assessed in all participants using the Epworth Sleepiness Scale (ESS).^[11]^ The second stage was a confirmatory case–control study that was conducted using PSG. Obstructive breathing events during monitored sleep were described according to the recommendation of the American Association of Sleep Medicine.^[12]^ The average number of apnea and hypopnea events per hour of sleep (i.e., the AHI score) was then calculated. Participants with an AHI score ≥ 5 were categorized as having OSA.

Successful PSG studies were conducted for only 346 participants, of whom 235 had OSA.^[3]^ The overall prevalence of OSA in the screened population was estimated to be at least 8.8%, affecting 12.8% of men and 5.1% of women.

In the present study, the PSG data of 235 patients with OSA were reviewed, and those with e-POSA were identified based on the criteria below. The prevalence of e-POSA in the PSG group was subsequently calculated. Then, the overall prevalence among the screened population was estimated following the same steps described in the original epidemiological study,^[3]^ as follows: the number of participants with e-POSA per PSG finding/total number of participants in the screened population × 100%.

### 2.2. Definitions of POSA

POSA is defined as an AHI score in the supine position at least twice as high as that in the lateral position, with e-POSA defined as an AHI score in the lateral position not exceeding 5.^[8]^ Participants with <240 minutes of total sleep time during PSG and those who spent <30 minutes in the supine or nonsupine position were excluded from the analysis.

### 2.3. Statistical analysis

The study presents findings from a dataset of 346 patients who underwent standard polysomnography from the Saudi population-based cohort. The dataset had missing values (2.1%). The multiple imputation method failed to impute missing values for some variables because of the skewness. Therefore, the single imputation regression method was used, which employs estimation based on linear regression for parametric variables, and logistic regression for the categorical variables. The imputed dataset had negative values, and these were rounded to zero because none of the study variables were expected to have negative values. All categorical variables were rounded to the nearest whole number. All the descriptive and inferential statistics were performed on the imputed dataset. For group comparison, the Chi-square test, and Fisher exact test were used for the categorical variables. For group comparison, an independent *t* test was used for the continuous variables where assumptions of normal distribution across both groups were satisfied. For group comparison, the Mann–Whitney test was used for the continuous variables, for which, assumptions of normal distribution across both groups were not satisfied.

### 2.4. Assumption testing for the binary logistic regression

The selection of the predictors was based on both theoretical and statistical considerations. Male sex, a body mass index (BMI) <35 kg/m^2^, younger age, mild disease characterized by an AHI score <10 and an AHI-rapid eye movement (REM) score <20, duration of sleep with an oxygen saturation <90%, and a history of diabetes, hypertension, and asthma have been associated with e-POSA.^[6,9]^ Therefore, in the present study, we also selected sex, BMI, age, AHI score, AHI-REM score, duration of sleep with an oxygen saturation <90%, and a history of diabetes, hypertension, and asthma as predictors in the model. Moreover, we identified some predictors based on the rule of thumb criteria, i.e., a bivariate correlation coefficient of more than 0.2 and/or a *P* value less than.05. Based on this statistical consideration, we identified the REM duration, number of all desaturations below 90%, AHI score in the supine position, AHI score in the nonsupine position, AHI score, and total sleep time in the supine position as additional predictors. Binary logistic regression was performed with the dichotomous variable of e-POSA (yes/no) and the abovementioned predictors. No issues of multicollinearity were found, with tolerance values for all predictor variables being >0.1 and all variance inflation factors (VIFs) being <10. The number of all desaturations below 90% and the sum of all desaturations (%) were decreased because of multicollinearity issues, as indicated by interpredictor correlations above 0.7. Twenty-seven outlier cases were identified based on the Mahalanobis distance (Χ2 (14) = 29.14, *P* < .01). However, all the cases were retained in the model after verifying that the values for the individual variables in seventeen cases were within the clinical range. All the continuous predictor variables satisfied the linearity test. A *P* value of.05 was considered to indicate statistical significance. All the tests were performed in SPSS version 23.0.

## 3. Results

### 3.1. General characteristics of the participants in the Saudi population-based cohort

The participants’ general characteristics are shown in Table 1. The average age, neck circumference, waist circumference, hip circumference, and waist-to-hip ratio were 42.36 ± 6.68 years, 35.75 ± 4.01 cm, 95.64 ± 12.79 cm, 107.96 ± 11.99 cm, and 0.89 ± 0.08, respectively. Most participants were male (66.5%) and had mild to severe sleep apnea (67.9%). Approximately 1/3 of the participants (28.6%) experienced daytime sleepiness and had an ESS score >10. More than half of the participants had obesity (52.6%), with a BMI of 30 kg/m^2^ or above. The prevalence of hypertension, diabetes mellitus, asthma, and chronic bronchitis was 18.5%, 11.8%, 15.9%, and 4.3%, respectively. More than 1/5 of the participants (20.2%) were current smokers, while 11.8% reported being ex-smokers.

**Table 1:** General characteristics of the participants who underwent a sleep study (n = 346).

Variables	Mean ± SD/number (%)
Ages (yr)	42.36 ± 6.68
Sex Male Female	230 (66.5) 116 (33.5)
Hypertension No Yes	282 (81.5) 64 (18.5)
Diabetes No Yes	305 (88.2) 41 (11.8)
Asthma No Yes	291 (84.1) 55 (15.9)
Chronic Bronchitis No Yes	331 (95.7) 15 (4.3)
Current smoking No Yes	276 (79.8) 70 (20.2)
Ex-smoking No Yes	304 (87.9) 41 (11.8)
Neck circumference	35.75 ± 4.01
Waist circumference	95.64 ± 12.79
Hip circumference	107.96 ± 11.99
Waist-hip ratio	0.89 ± 0.08
Obesity (BMI ≥ 30 kg/m^2^) No Yes	164 (47.4) 182 (52.6)
ESS ESS score ≤ 10 ESS score > 10	247 (71.4) 99 (28.6)
OSA AHI score < 5 AHI score ≥ 5	111 (32.1) 235 (67.9)

AHI = apnea-hypopnea index, BMI = body mass index, ESS = Epworth Sleepiness Scale, OSA = obstructive sleep apnea, SD = standard deviation.

### 3.2. Prevalence of e-POSA in OSA patients and the Saudi Arabian population

**Figure 1.**
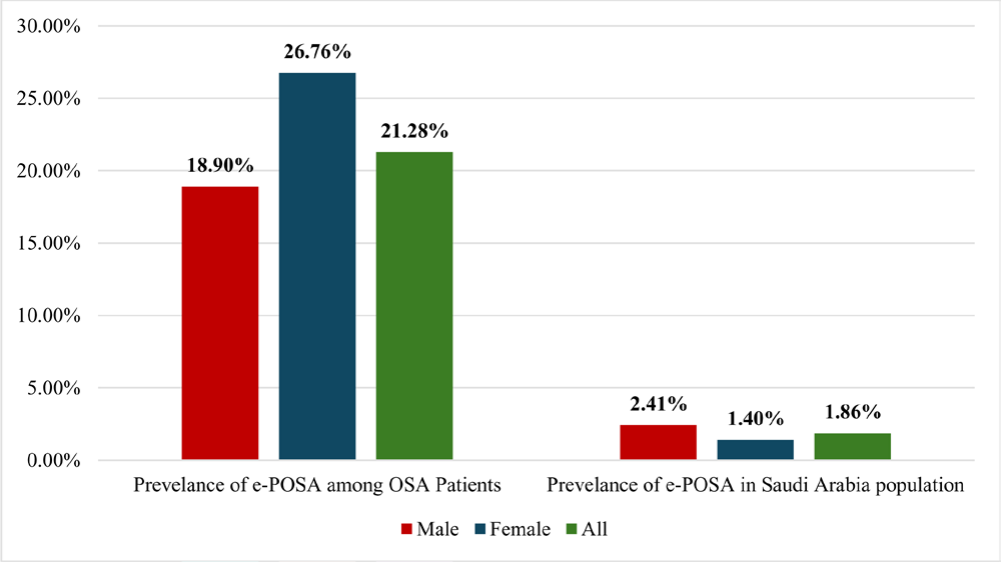
Prevalence of positional obstructive sleep apnea (e-POSA) in OSA patients and the Saudi Arabian population.

Figure 1 shows a significant prevalence of e-POSA in OSA patients (21.28%), with a greater prevalence in females (26.76%) than in males (18.90%). When extrapolated to the screened population, the prevalence of e-POSA was estimated to be 1.86%, with a higher prevalence in males (2.41%) than in females (1.40%).

### 3.3. General and apnea-related characteristics among sleep apnea patients

e-POSA patients accounted for 14.45% of patients who underwent PSG and 21.28% of all patients who had OSA. The average age, BMI, ESS score, neck circumference, waist circumference, hip circumference, waist-to-hip ratio, AHI score, mixed apnea index score, central apnea index score, obstructive apnea index score, obstructive hypoapnea index score, duration of sleep with an oxygen saturation < 90%, mean O_2_ saturation, minimum O_2_ saturation, periodic limb movement (PLM) index score, arousal index score, PLM arousal index score and respiratory arousal index score were 42.85 ± 6.77 years, 30.05 ± 5.64 kg/m^2^, 7.27 ± 5.56, 35.82 ± 4.05 cm, 95.12 ± 12.74 cm, 107.50 ± 11.36 cm, 0.88 ± 0.08, 18.45 ± 14.88 events/hour, 0.06 ± 0.33 events/hour, 0.38 ± 3.10 events/hour, 2.92 ± 8.10 events/hour, 15.09 ± 10.95 events/hour, 5.61 ± 15.96 minutes, 0.95 ± 0.02, 0.85 ± .07, 4.41 ± 8.84 events/hour, 23.15 ± 12.72 events/hour, 0.47 ± 1.13 events/hour, and 8.76 ± 9.12 event/hour, respectively (Table 2). The prevalence rates of obesity, hypertension, diabetes mellitus, asthma, and chronic bronchitis were 46.8%, 23%, 14.5%, 16.6%, and 4.3%, respectively. More than 1/5 of the participants (21.7%) were current smokers, while 13.6% reported being ex-smokers (Table 2).

**Table 2:** Characteristics of OSA patients: a comparison of the non-POSA and e-POSA groups.

Variables	OSA (n = 235)	Non e-POSA (n = 185)	e-POSA (n = 50)	*P* value
**Ages (yr**)	**42.85 ± 6.77**	**43.31 ± 6.81**	**41.15 ± 6.39**	**.05** *
Sex Male Female	164 (69.8) 71 (30.2)	133 (71.9) 52 (28.1)	31 (62.0) 19 (38.0)	.18
BMI	30.05 ± 5.64	30.05 ± 5.79	30.04 ± 5.12	.84†
Obesity (BMI ≥ 30 kg/m^2^) No Yes	125 (53.2) 110 (46.8)	95 (51.4) 90 (48.6)	30 (60.0) 20 (40.0)	.28
**ESS (mean**)	**7.27 ± 5.56**	**8.01 ± 5.73**	**4.52 ± 3.87**	**<.01** †
**Excessive daytime sleepiness (ESS score > 10**) **No** **Yes**	**152 (64.7**) **83 (35.3**)	**108 (58.4**) **77 (41.6**)	**44 (88.0**) **6 (12.0**)	**<.01**
Hypertension No Yes	181 (77.0) 54 (23.0)	140 (75.7) 45 (24.3)	41 (82.0) 9 (18.0)	.35
Diabetes No Yes	201 (85.5) 34 (14.5)	159 (85.9) 26 (14.1)	42 (84.0) 8 (16.0)	.73
Asthma No Yes	196 (83.4) 39 (16.6)	152 (82.2) 33 (17.8)	44 (88.0) 6 (12.0)	.32
Chronic bronchitis No Yes	225 (95.7) 10 (4.3)	178 (96.2) 7 (3.8)	47 (94.0) 3 (6.0)	.45
Current smoker No Yes	184 (78.3) 51 (21.7)	142 (76.8) 43 (23.2)	42 (84.0) 8 (16.0)	.27
Ex-smoker No Yes	203 (86.4) 32 (13.6)	158 (85.4) 27 (14.6)	45 (90.0) 5 (10.0)	.40
Neck Circumference	35.82 ± 4.05	35.96 ± 4.02	35.30 ± 4.13	.15†
	95.12 ± 12.74	95.58 ± 12.94	93.43 ± 11.95	.30†
	107.50 ± 11.36	107.49 ± 11.71	107.56 ± 10.03	.96†
	0.88 ± 0.08	0.89 ± 0.08	0.87 ± 0.08	.08†
**AHI**	**18.45 ± 14.88**	**20.28 ± 15.84**	**11.67 ± 7.46**	**<.01** †
Mixed apnea index	0.06 ± 0.33	0.07 ± 0.37	0.01 ± 0.05	.21†
Central apnea index	0.39 ± 3.15	0.48 ± 3.54	0.07 ± 0.24	.251
Obstructive apnea index	2.92 ± 8.10	3.06 ± 8.68	2.38 ± 5.42	.42†
**Obstructive hypopnea index**	**15.09 ± 10.95**	**16.71 ± 11.58**	**9.10 ± 4.76**	**<.01** †
Time spent < 90% O_2_ saturation	5.61 ± 15.96	6.38 ± 17.25	2.74 ± 9.36	.38†
Mean O_2_ saturation	0.95 ± 0.02	0.95 ± 0.03	0.95 ± 0.02	.54†
Minimum O_2_ saturation	0.85 ± .07	0.85 ± 0.07	0.86 ± 0.05	.53†
PLM index	4.41 ± 8.84	4.80 ± 9.58	2.97 ± 5.06	.43†
**Arousal index**	**23.15 ± 12.72**	**24.40 ± 13.05**	**18.54 ± 10.31**	**<.01** †
PLM arousal index	0.47 ± 1.13	0.50 ± 1.22	0.36 ± 0.71	.91†
**Respiratory arousal index**	**8.76 ± 9.12**	**9.87 ± 9.80**	**4.66 ± 3.89**	**<.01** †

*P* value < .005 is considered significant.

AHI = apnea-hypopnea index, BMI = body mass index, e-POSA = exclusive positional obstructive sleep apnea, ESS = Epworth Sleepiness Scale, OSA = obstructive sleep apnea, PLM = periodic limb movement.

* Independent *t* test.

† Mann–Whitney test.

### 3.4. General and apnea-related characteristics: comparison between patients with e-POSA and those with nonpositional sleep apnea (non-POSA)

The e-POSA group tended to be younger (41.15 ± 6.39 years) than the non-POSA group (43.31 ± 6.81 years; *P* = .05). Additionally, compared with non-e-POSA patients, e-POSA patients had a significantly lower ESS score (8.01 ± 5.73 and 4.52 ± 3.87, respectively; *P* < .001). In addition, the AHI score was significantly greater in patients with non-e-POSA (20.28 ± 15.84) than in those with e-POSA (11.67 ± 7.46; *P* < .001). Similarly, the obstructive-hypopnea index (*P* < .001), arousal index (*P* < .001), and respiratory arousal index (*P* < .001) scores were significantly greater in patients with non-e-POSA (Table 2).

### 3.5. Factors that determine e-POSA

Table 3 shows the results of the binary logistic regression to identify predictors of e-POSA. The model was adjusted for sex, BMI, age, AHI score, AHI-REM score, duration of sleep with an oxygen saturation <90%, a history of diabetes, hypertension, and asthma, REM duration, the number of all desaturations, the number of all desaturations below 90%, the sum of all desaturations (time), the sum of all desaturations (%), the AHI-supine score by the AHI-nonsupine score, the AHI-supine score, the AHI-nonsupine score, the AHI score, and total sleep time in the supine position. The binary logistic model predicted 39.9% of the variance in classifying patients with e-POS, (χ^2^[16, N = 346] = 87.02, *P* < .001). e-POSA was associated with an increased AHI score during REM sleep (adjusted odds ratio [AOR] 1.025, 95% confidence interval [CI] 1.006–1.044, *P* = .01) and a decreased AHI score during nonsupine sleep (AOR 0.825, 95% CI 0.753–0.904, *P* < .001).

**Table 3:** Predictors of Positional Obstructive Sleep Apnea: A Multivariate Logistic Regression Analysis.

Predictors	*B*	AOR (95% CI)	*P* value
Age	0.001	1 (0.94–1.06)	.95
Sex	0.14	1.15 (0.52–2.56)	.73
Obesity	0.01	1.01 (0.47–2.18)	.98
Hypertension	0.74	2.1 (0.59–7.41)	.25
Diabetes mellitus	−1.8	0.16 (0.02–1.14)	.07
Asthma	−0.71	0.49 (0.15–1.58)	.23
Total AHI	−0.05	0.95 (0.88–1.02)	.15
Duration of sleep with an oxygen saturation < 90%	−0.02	0.98 (0.94–1.02)	.26
Number of all desaturations below 90%	−0.02	0.98 (0.95–1.01)	.2
REM duration (min)	0.01	1.01 (1–1.03)	.18
**AHI during REM**	**0.01**	**1.025 (1.006–1.044**)	**.01**
AHI during supine sleep	0.001	1.02 (0.96–1.08)	.51
**AHI during non-supine sleep**	**−0.27**	**0.825 (0.753–0.904**)	**<.001**
Total supine sleep time (in min)	0.01	1.01 (1.00–1.01)	.25

Age, sex, and obesity status were dichotomized as BMI (≥30 kg/m^2^) variables; hypertension history, diabetes mellitus status, asthma status, total AHI, and duration of sleep with an oxygen saturation <90% were dichotomized (>14.2 minutes); and the number of all desaturations below 90%, REM duration (min), AHI during REM, AHI during nonsupine sleep, AHI during supine sleep, and the total sleep duration (min) were dichotomized. *P* value < .005 is considered significant.

AHI = apnea hypoapnea index, AOR = adjusted odds ratio, *B* = *B* coefficient, CI = confidence interval, COR = crude odds ratio, REM = rapid eye movement.

## 4. Discussion

In our study of 346 participants assessed for OSA through PSG, 235 patients with OSA were identified (AHI score ≥ 5). However, the prevalence of e-POSA in OSA patients was 21.28%, with a higher prevalence in females (26.76%) than in males (18.90%). Moreover, the estimated prevalence of e-POSA among the general population was 1.86%. Moreover, the e-POSA patients were significantly younger, had lower AHI, ESS, and arousal index scores and experienced less daytime sleepiness (*P* < .001). Furthermore, an increase in the AHI-REM score and a decrease in the AHI-nonsupine score were identified as predictors of POSA using multivariate logistic regression analysis.

In our study, we diagnosed e-POSA in approximately one-fifth (21.28%) of the OSA patients, who were significantly younger, with a notable predominance of females compared to males. This pattern aligns with previous studies investigating the prevalence of e-POSA, age and the sex distribution,^[9,13–16]^ with the prevalence ranging from 20.1% to 33.8%. Moreover, Heinzer et al^[9]^ evaluated 1224 OSA patients in Switzerland and reported that the prevalence of e-POSA was 36%, with a significant predominance of females and greater susceptibility among younger individuals. Furthermore, our study estimated a e-POSA prevalence rate of 1.86% among the Saudi population, which represents a novel contribution to the field. To our knowledge, there is only 1 study in which the prevalence of e-POSA in the general population has been estimated.^[9]^ However, our finding differs significantly from the prevalence reported by Heinzer et al^[9]^ (26%). This discrepancy may be attributed to the difference in the methods employed to estimate the prevalence, the demographic dissimilarities between the populations studied and the remarkably high OSA prevalence in the general population reported in the study by Heinzer et al^[9]^ compared to other similar epidemiological studies.^[3,17]^ The latter would inevitably increase the percentage of e-POSA in the studied population.

Furthermore, our study highlighted a significantly lower AHI score in the e-POSA group than in the non-POSA group, indicating that e-POSA is predominantly associated with milder cases of OSA. This finding is in line with previous research,^[8,9,13,18,19]^ where it has consistently been demonstrated that individuals with e-POSA tend to have a lower AHI score, suggesting a milder form of OSA. Recently, Stafford et al^[15]^ examined 215 OSA patients and identified e-POSA in 24% of the patients, with a distinct frequency in patients with mild OSA compared with those with moderate or severe OSA (28% vs 20% vs 17%, respectively).

Moreover, our results also demonstrated that e-POSA patients had a significantly lower respiratory arousal index and total arousal index scores. This aligns with findings in the literature.^[13,20]^ Oksenberg et al^[13]^ analyzed polysomnographic data from 574 patients who underwent PSG and found that compared with non-POSA patients, POSA patients exhibited significantly fewer arousals without any significant differences in the number of total limb movements or the number of limb movements causing arousal. This lower incidence of arousals in POSA patients may contribute to a less fragmented sleep architecture, potentially translating into better overall sleep quality and, consequently, better daytime functioning. This is further supported by the fact that our e-POSA patients had less daytime sleepiness than non-POSA patients (*P* < .001). These findings are in line with those of Huang et al,^[21]^ who reported that POSA patients are less likely to feel sleepy and experience EDS. Overall, these findings and the lower AHI score support the notion that patients with e-POSA tend to have milder sleep apnea.

In addition, our investigation did not reveal a significant correlation between e-POSA and lower BMI values. In contrast, several studies have established this relationship.^[6,13,15,16]^ This discrepancy may be due to the high prevalence of obesity within our study cohort, with 52.6% of the participants classified as obese. The substantial proportion of obese individuals could obscure the potential association between lower BMI values and the incidence of e-POSA, complicating the identification of such a link in our population. Although e-POSA patients tend to have a lower BMI, most treatment options are geared toward addressing the relationship among the AHI score, sleep position, and obesity. Consequently, weight loss strategies and positional therapy have been found to be effective in these patients. Given that weight loss and lateral positioning are both known to improve upper airway collapsibility, Joosten et al^[22]^ reported that the combined effect of weight loss and lateral positioning during sleep might normalize the AHI in a greater number of patients than weight loss alone. These findings suggest that an integrated approach involving weight management and positional therapy can significantly impact treatment outcomes for patients with e-POSA, suggesting a multidimensional treatment strategy that considers the complex interplay of various factors affecting OSA severity.

Our study further revealed that an increase in the AHI-REM and AHI-supine scores with a decrease in the AHI-nonsupine score were identified as significant predictors for e-POSA. The increase in the AHI-REM score as a predictor for ePOSA aligns with the findings from our previous study,^[6]^ in which an AHI-REM score > 20 was identified as a predictor for e-POSA.^[8]^ This relationship could be due to the remarkable decrease in muscle tone of the pharynx during REM sleep, increasing susceptibility to airway collapse. This subsequently enhances the likelihood of the tongue and soft tissues falling backward into the airway while in the supine position due to gravity.^[23]^ The other predictors, namely, the increase in the AHI-supine score with a decrease in the AHI-nonsupine score, are actually essential for the definition of e-POSA.

Despite the valuable insights provided by this study, certain limitations should be acknowledged. The sample predominantly composed of school employees with a high prevalence of obesity which may limit the generalizability of the findings to broader populations. Additionally, the extrapolation method used to estimate e-POSA prevalence in the general population may introduce potential inaccuracies. Future research should focus on more heterogeneous samples, incorporating participants from diverse professions and socioeconomic backgrounds, to enhance the generalizability and accuracy of the prevalence estimates. Furthermore, while this study identified AHI-REM and AHI-nonsupine as predictive factors, future studies should explore additional predisposing factors to provide a more comprehensive understanding of e-POSA.

In conclusion, our study is one of the few studies to estimate the prevalence of positional sleep apnea in the general population. This represents a significant contribution to the understanding of this condition. We found that e-POSA is present in approximately one-fifth (21.28%) of OSA patients, with a notable prevalence in females compared to males. POSA is associated with milder OSA, less daytime sleepiness and less symptoms with a lower arousal index score. Comorbidities were not found to be linked with POSA. However, regression analysis revealed that only an increase in the AHI-REM score and a decrease in the AHI-nonsupine score were identified as predictors for e-POSA. These findings offer valuable insights that can improve clinical practice and guide future research efforts in the field of sleep medicine, ultimately improving the diagnosis, management, and understanding of positional sleep apnea globally.

## Author contributions

**Conceptualization:** Siraj Wali, Faris Alhejaili, Ranya Alshumrani, Haneen Mansour.

**Data curation:** Siraj Wali, Omar Kanbr, Faris Alhejaili, Ranya Alshumrani, Md Dilshad Manzar, Haneen Mansour.

**Formal analysis:** Siraj Wali, Md Dilshad Manzar.

**Funding acquisition:** Siraj Wali.

**Investigation:** Siraj Wali, Omar Kanbr, Ranya Alshumrani.

**Methodology:** Siraj Wali, Omar Kanbr, Ranya Alshumrani, Haneen Mansour.

**Project administration:** Siraj Wali.

**Resources:** Siraj Wali.

**Writing – original draft:** Siraj Wali, Omar Kanbr, Faris Alhejaili, Ranya Alshumrani, Md Dilshad Manzar, Haneen Mansour.

**Writing – review & editing:** Siraj Wali, Faris Alhejaili, Ranya Alshumrani, Md Dilshad Manzar.
